# Identification of poly(I:C) interacted proteins and their regulation in transcription level against bacterial infection in zebrafish

**DOI:** 10.3389/fimmu.2026.1848091

**Published:** 2026-07-03

**Authors:** Hui-yin Lin, Jiao Xiang, Yi Han, Xuan-xian Peng, Hui Li, Xian-jie Liu

**Affiliations:** 1 State Key Laboratory of Bio-Control, School of Life Sciences, Southern Marine Science and Engineering Guangdong Laboratory (Zhuhai), Guangdong Province Key Laboratory for Pharmaceutical Functional Genes, Sun Yat-sen University, University City, Guangzhou, China; 2 Shenzhen International Institute for Biomedical Research, Shenzhen, China

**Keywords:** affinity-proteomics, bacterial infection, poly(I:C), protein-protein interaction (PPI), zebrafish

## Abstract

**Introduction:**

Polyinosinic-polycytidylic acid (poly(I:C)), a synthetic double-stranded RNA (dsRNA) analog, activates innate immunity against infectious and non-infectious diseases through interactions with both DNA and proteins. However, the full spectrum of poly(I:C)-binding proteins and their roles remains unclear.

**Methods:**

Affinity-proteomics was used to identify poly(I:C)-interacting proteins in zebrafish, with four candidates validated by microscale thermophoresis (MST). Gene expression of all 27 targets was analyzed by qRT-PCR in poly(I:C)-treated fish and in fish surviving or dying after *Vibrio alginolyticus* or *Edwardsiella tarda* challenge.

**Results:**

Twenty-seven poly(I:C)-interacting proteins involved in diverse cellular processes were identified. Four candidatesprolyl 4-hydroxylase (P4HTM), acidic leucine-rich nuclear phosphoprotein 32 family member E (ANP32E), F-box only protein 2 (FBXO2), and ribosomal protein large P2 (RPLP2)were validated for binding via microscale thermophoresis, with P4HTM's functional modulation by poly(I:C) further demonstrated. At the gene expression level, 10 targets were upregulated, 9 of which showed elevated levels in surviving fish but declined in dying fish after *Vibrio alginolyticus* or *Edwardsiella tarda* challenge, implicating them as anti-infective biomarkers

**Discussion:**

These findings reveal poly(I:C)'s broader protein targets than previously recognized, offering new insights into its multifaceted biological functions in antibacterial defense in fish, with broader implications for understanding evolutionarily conserved dsRNAhost interactions in vertebrate innate immunity.

## 1 Introduction

Polyinosinic-polycytidylic acid [poly(I:C)] is a synthetic double-stranded RNA (dsRNA) molecule formed by annealing polyinosinic acid and polycytidylic acid homopolymers (1). It elicits robust innate immune responses, conferring protection against viral and bacterial infections (2, 3) and serving as a therapeutic agent in cancer treatment (4), neuroprotection, and post-ischemic brain recovery (5). These effects are mediated through pattern-recognition receptors (PRRs), including the membrane-bound Toll-like receptor 3 (TLR3) and cytosolic RIG-I-like receptors such as retinoic acid-inducible gene I (RIG-I) and melanoma differentiation-associated protein 5 (MDA5) (6). PRR activation triggers signaling cascades involving NF-κB, MAP kinases, and interferon regulatory factors (IRFs), leading to the production of type I interferons and inflammatory cytokines that combat pathogens and modulate disease (2, 7, 8). Poly(I:C) can also directly induce apoptosis in TLR3-expressing cancer cells (4) and influence metabolic pathways, including glucose homeostasis and central carbon metabolism via TCA cycle enhancement (9, 10), as well as lipid metabolism (11).

While extensively studied in mammalian contexts, poly(I:C) plays a critical role in aquaculture by activating PRRs (12), initiating the JAK-STAT antiviral pathway (13), and upregulating immune-related genes such as small heat shock proteins (sHSPs), poly(I:C)-inducible protein 1 (PIP1), and C1q-domain-containing proteins (C1qDCs) in *Crassostrea gigas* (14). Additionally, it modulates metabolism-related miRNAs in gibel carp kidney tissue (15). Our recent work revealed a novel mechanism by which poly(I:C) enhances the TCA cycle to strengthen innate immunity against *Vibrio alginolyticus* via malate (10), suggesting interactions with non-PRR targets to exert broader biological effects.

However, these targets remain largely unidentified. Elucidating them is essential for a comprehensive understanding of poly(I:C)’s mechanisms. Since the metabolic effects observed in our previous study (10) are difficult to attribute solely to canonical PRR signaling, we hypothesized the existence of direct, non-canonical poly(I:C)-binding proteins. To investigate whether the identified poly(I:C)-interacting proteins contribute to anti-infective immunity, we employed adult zebrafish (*Danio rerio*) — a widely used vertebrate model with conserved innate immune pathways — and challenged fish with *V. alginolyticus* and *E. tarda*, two economically significant aquaculture pathogens responsible for vibriosis and edwardsiellosis in commercially farmed marine and freshwater species, respectively. This study therefore employed a resin-immobilized poly(I:C) proteomics approach to identify its target proteins in this model system.

## 2 Materials and methods

### 2.1 Protein extraction from zebrafish

Adult zebrafish (husbandry details provided in Section 2.10) were euthanized by immersion in 300 mg/L tricaine methanesulfonate (MS-222; Sigma-Aldrich) at least 10 minutes after cessation of opercular movement. Following surface drying with filter paper, whole zebrafish bodies were placed in a pre-chilled mortar and pulverized into a fine powder under liquid nitrogen. The resulting powder was homogenized in lysis buffer (containing 40 μL RIPA, 5 μL protease inhibitor, 5 μL phosphatase inhibitor, and 0.5 μL PMSF) for 30 min. Cellular debris was removed by centrifugation at 12,000 × g for 20 min at 4 °C. The protein concentration of the supernatant was quantified using the Bradford assay. Whole-body protein extracts were pooled from five adult zebrafish per preparation, and three independent biological replicates were performed. After which aliquots were prepared and stored at -20 °C until further analysis.

### 2.2 Affinity-proteomics identification of poly(I:C)-binding proteins

The poly(I:C)-binding proteins were isolated using an affinity-proteomics approach adapted from Zhao et al. (16). Briefly, epoxy-activated Sepharose 6B (Sigma-Aldrich) was employed as the solid support for poly(I:C) immobilization. Approximately 300 mg of freeze-dried epoxy-activated Sepharose 6B was rehydrated in deionized water and washed thoroughly to obtain the wet medium. The resin was then incubated with 500 μM poly(I:C) in coupling buffer (0.1mol/L NaHCO3, 0.5 mol/L NaCl, pH13) at 37 °C for 16 h with gentle agitation. Following coupling, the medium was washed three times with coupling buffer, and remaining active sites were blocked with 1 M ethanolamine (pH 8.0). For protein binding, zebrafish protein extracts were loaded onto the poly(I:C)-conjugated affinity column. Unbound proteins were removed by extensive washing with PBS (50 mM, pH 7.0) until the A280 of the effluent reached baseline levels. Specifically bound proteins were eluted with 3 mL of 0.1 M glycine-HCl (pH 2.4) and immediately neutralized with 300 μL of 1 M Tris-HCl (pH 8.0). The eluted proteins were concentrated by cold acetone precipitation (-20 °C), resolved by SDS-PAGE, and subsequently transferred to a nitrocellulose membrane for immunoblotting analysis.

### 2.3 LC-MS sample preparation procedure, iTRAQ labeling, and proteomics analysis

An 80 µL aliquot of the affinity-purified eluate obtained from Section 2.2 (poly(I:C)-bound proteins eluted with 0.1 M glycine-HCl, pH 2.4, and neutralized with 1 M Tris-HCl, pH 8.0) was mixed with 5 µL of 50 mM NH_4_HCO_3_ containing 250 mM dithiothreitol (DTT) and incubated at 55 °C for 1 hour. Following reduction, 5 µL of 50 mM NH_4_HCO_3_ containing 625 mM iodoacetamide (IAA) was added, and the mixture was incubated at room temperature in the dark for 1 hour for alkylation. Digestion was performed by adding trypsin solution at an enzyme-to-protein ratio of 1:50 (w/w) and incubating at 37 °C overnight. The resulting peptide mixture was concentrated to approximately 20 µL using a vacuum concentrator. To remove insoluble particulates, the concentrated sample was centrifuged at 12,000 × g for 10 minutes; this step was repeated once.

Isobaric tags for relative and absolute quantification (iTRAQ) labeling of peptide samples was performed using an iTRAQ Reagent kit (AB SCIEX, USA) according to the manufacturer’s protocol. Duplicate samples of the iTRAQ-labeled peptides were concentrated to approximately 20 µL using a vacuum centrifuge. The labeled samples were desalted using a Strata-X 33 µm polymeric reversed-phase column (Phenomenex, USA). The purified peptides were resuspended in buffer A (5% acetonitrile, 0.1% formic acid) and analyzed using an AB SCIEX Triple-TOF 5600 mass spectrometer coupled with a Nanospray III source and a pulled quartz tip.

Raw data files (.wiff) were converted to.mgf format using AB SCIEX MS Data Converter V1.1 software. Protein identification and quantification were performed using ProteinPilot™ Software 4.5 with the following parameters: Sample Type, iTRAQ 4-plex (Peptide Labeled); Cysteine Alkylation, Iodoacetamide; Digestion, Trypsin; Instrument, Triple-TOF 5600; ID Focus, Biological modifications; Database, Danio_rerio.GRCz11.dna.toplevel.fa; Search Effort, Thorough. The detection threshold was set at Unused ProtScore (Conf) > 1.30 (95.0% confidence). The false discovery rate was estimated using a reverse database search strategy. All proteins were identified by ≥ 2 unique peptides.

### 2.4 Bioinformatics analysis of protein-protein interactions

The 27 zebrafish proteins interacting with poly(I:C) were functionally annotated using the UniProt database (http://www.uniprot.org/) to determine their subcellular localization, molecular functions, and associated biological processes. Subsequently, protein-protein interaction networks were constructed for these proteins using the STRING database (http://string-db.org/) (17), with their UniProt IDs serving as input.

### 2.5 Cloning of genes and recombinant protein expression

Gene cloning and recombinant protein expression were performed following established protocols (18). Briefly, approximately 100 mg of zebrafish tissue was flash-frozen in liquid nitrogen and homogenized in a pre-chilled mortar. Total RNA was extracted using a commercial RNA extraction kit, and its purity was assessed by measuring the absorbance at 260 nm and 280 nm (A260/A280 ratio). RNA integrity was verified by 1% agarose gel electrophoresis. High-quality RNA samples were stored at –80 °C for subsequent experiments. Zebrafish protein gene sequences were retrieved from the UniProt database, and gene-specific primers (**Supplementary Table 1**) were designed and synthesized by Shenzhen Huada Gene Technology Co., Ltd. The recombinant proteins were expressed with an N-terminal thioredoxin (Trx) tag (approximately 20 kDa) to enhance solubility.

### 2.6 Microscale thermophoresis assay

MST, a technique for quantifying biomolecular interactions, assay was performed as previously described (19, 20). Briefly, target proteins were fluorescently labeled using the Monolith NT Protein Labeling Kit Red-NHS (#MO-L011, NanoTemper Technologies, Munich, Germany) in MST-optimized buffer according to the manufacturer’s protocol. Proteins (targets) were incubated with 50 μM dye for 30 min at room temperature, followed by a 5 min incubation with poly(I:C) or interacting proteins (ligands). Binding reactions were loaded into premium-coated glass capillaries, and MST measurements were conducted on a Monolith NT.115 instrument (NanoTemper Technologies) at 40% MST power (infrared laser) and 40% LED power (excitation light). Data were analyzed using MO. Affinity Analysis software (NanoTemper Technologies), with the Thermophoresis + T-Jump signal used for quantification. Three independent replicates were performed for each interaction. Dose-response curves were fitted using GraphPad Prism software (version 5.0, GraphPad Software Inc.) to determine binding affinities.

### 2.7 Effect of poly(I:C) on prolyl hydroxylase activity

To investigate whether poly(I:C) binding to P4HTM inhibits its prolyl hydroxylase activity toward the HIF-1α ODD domain, reaction mixtures (10 μL total volume) containing 2 μg recombinant oxygen-dependent degradation (ODD) domain protein (cloned and expressed as described in Section 2.5), 3.5 μg recombinant prolyl hydroxylase (PHD), and different concentrations of poly(I:C) (1, 2, 3, and 4 μg) were prepared in reaction buffer consisting of 20 mM Tris-HCl (pH 7.4), 5 mM KCl, 1.5 mM MgCl_2_, 1 mM DTT, 5 mM sodium ascorbate, 0.1 mM FeCl_2_, and 1 mM 2-oxoglutarate. The reactions were incubated at 37 °C for 15 min and then analyzed by SDS-PAGE. Western blot analysis was performed using an antibody against the ODD domain protein as the primary antibody. The antibody used for Western blot analysis was generated in-house using the same recombinant HIF-1α ODD protein described in Section 2.5. It is a mouse polyclonal antibody raised against the purified recombinant ODD protein. The serum preparation procedures are described in detail in the Section 2.8.

### 2.8 Antiserum preparation

Four- to six-week-old BALB/c mice (weighing 20 ± 2 g) were obtained from the Animal Center of Sun Yat-sen University. The mice were housed in cages and provided with sterile water and a standard laboratory diet *ad libitum*. All mouse experiments were approved by the Sun Yat-sen University Institutional Animal Care and Use Committee (SYSU-IACUC-2022-B0112). Briefly, mice were immunized with 100 μg of recombinant prolyl 4-hydroxylase transmembrane (P4HTM), acidic leucine-rich nuclear phosphoprotein 32 family member E (ANP32E), F-box only protein 2 (FBXO2), or ribosomal protein large P2 (RPLP2) (selected as representatives of distinct functional categories of the poly(I:C) interactome based on affinity-proteomics and bioinformatics analyses; see Section 3.3 for details) emulsified in Freund’s complete adjuvant for the primary immunization, followed by booster injections with the same amount of antigen in Freund’s incomplete adjuvant at 7-day intervals. Following immunization, blood samples were collected via orbital bleeding while mice were anesthetized with sodium pentobarbital (50 mg/kg, intraperitoneal injection) as a survival procedure to monitor antiserum titer development by Western blot analysis. Post-procedural monitoring was performed to ensure full recovery. At the experimental endpoint, mice were first deeply anesthetized by intraperitoneal injection of sodium pentobarbital (50 mg/kg). While still under deep anesthesia, euthanasia was then performed by cervical dislocation. Death was verified by the absence of heartbeat and respiration for a minimum of 5 minutes.

### 2.9 Western blot analysis

Western blot analysis was performed according to established methods (21). Protein samples from zebrafish were separated by 10% SDS-PAGE and subsequently transferred to nitrocellulose membranes (GE Healthcare Life Sciences). The membranes were probed overnight at 4 °C with the following primary antibodies: mouse anti-P4HTM (1:500), anti-RPLP2 (1:500), anti-FBXO2 (1:500), and anti-ANP32E (1:500), or rabbit anti-β-actin (1:500) as loading control. After washing, membranes were incubated with appropriate horseradish peroxidase (HRP)-conjugated secondary antibodies (goat anti-rabbit or rabbit anti-mouse) for 1 h at room temperature. Protein bands were visualized using enhanced chemiluminescence and quantified with a CHEMI XT4 imaging system (Syngene).

### 2.10 Zebrafish challenge and specimen collection

Adult zebrafish (*Danio rerio*; ~0.2 g) obtained from a fish farm in Guangzhou were acclimated for two weeks in a laboratory recirculating aquaculture system (25 ± 1 °C, pH 7.5 ± 0.2, 12:12 light:dark cycle). Prior to bacterial challenge, zebrafish were anesthetized by immersion in buffered MS-222 (150 mg/L) until loss of equilibrium and response to touch. Bacterial challenges were conducted using stationary-phase cultures of *V. alginolyticus* 12G01 (cultured in 3% NaCl-supplemented LB at 30 °C) and *E. tarda* EIB202 (grown in TSB at 30 °C). Bacterial suspensions were prepared by: (1) adjusting OD600 to 0.5 in fresh media, (2) centrifugation (4,000×g, 10 min), (3) washing twice with appropriate saline (3% NaCl for 12G01; 0.85% NaCl for EIB202), and (4) diluting to predetermined LD50 concentrations (2× or 50× dilutions of OD600 = 1.0 suspensions). LD50 refers to the dose of bacteria that can kill half of the total experimental population. The LD50 concentration is first determined through preliminary dose-finding experiments and subsequently used as the challenge dose in the formal study. Experimental groups (n=20 fish/group) received intraperitoneal injections of 5 μL bacterial suspension (or sterile saline for controls) using 33-gauge needles. Mortality was monitored continuously, and moribund/deceased specimens were collected at 24 h for RNA isolation. All zebrafish experimental procedures were approved by the Sun Yat-sen University Institutional Animal Care and Use Committee (SYSU-IACUC-2022-B0112).

### 2.11 Quantitative real-time PCR analysis

qRT-PCR was performed following established protocols (22). Approximately 100 mg of zebrafish tissue was homogenized in liquid nitrogen using a pre-chilled mortar and pestle. Total RNA was isolated using TRIzol reagent (Ambion, Life Technologies) according to the manufacturer’s instructions. RNA quality and concentration were verified by spectrophotometry. First-strand cDNA was synthesized from 1 μg of total RNA using the PrimeScript RT reagent kit with gDNA Eraser (Takara Bio, Japan) to eliminate genomic DNA contamination. PCR amplification was performed in duplicate for each biological sample using the LightCycler 480 system (Roche Diagnostics, Germany) with SYBR Premix Ex Taq II (Takara Bio, Japan). The reaction conditions consisted of: initial denaturation at 95 °C for 30 sec; 40 cycles of 95 °C for 10 sec (denaturation), 60 °C for 30 sec (annealing), and 70 °C for 1 sec (fluorescence acquisition). A melting curve analysis was performed after amplification with a temperature ramp rate of 5 °C/sec from 65 °C to 95 °C. Gene-specific primer sequences are provided in (**Supplementary Table 2**). Expression of all 27 genes encoding poly(I:C)-binding proteins was analyzed: *apoa1, tpma, mylipa, calm2b, si:ch211-5k11.8, hbaa1, apoa2, c1qbp, vat1, crybb3, anp32e, skp1, ba1, actn3b, atp5a1, p4htm, zgc:92533, actb1, actb2, gnb1, fbxo2, tnni2a.3, cfl2l, lyz, zgc:56493, rplp2, and rplp2l*.Relative gene expression was calculated using the 2-ΔΔCt method with β-actin as the endogenous control. For poly(I:C) stimulation experiments, adult zebrafish were intraperitoneally injected with 56 μg poly(I:C) dissolved in 5 μL sterile saline; control fish received an equivalent volume of sterile saline. Fish were sampled at 24 h post-injection for RNA isolation and subsequent qRT-PCR analysis. 3 independent biological replicates (n = 3 fish per group) were used for all qRT-PCR analyses.

## 3 Results

### 3.1 Identification of poly(I:C)-binding proteins in zebrafish

**Figure 1:**
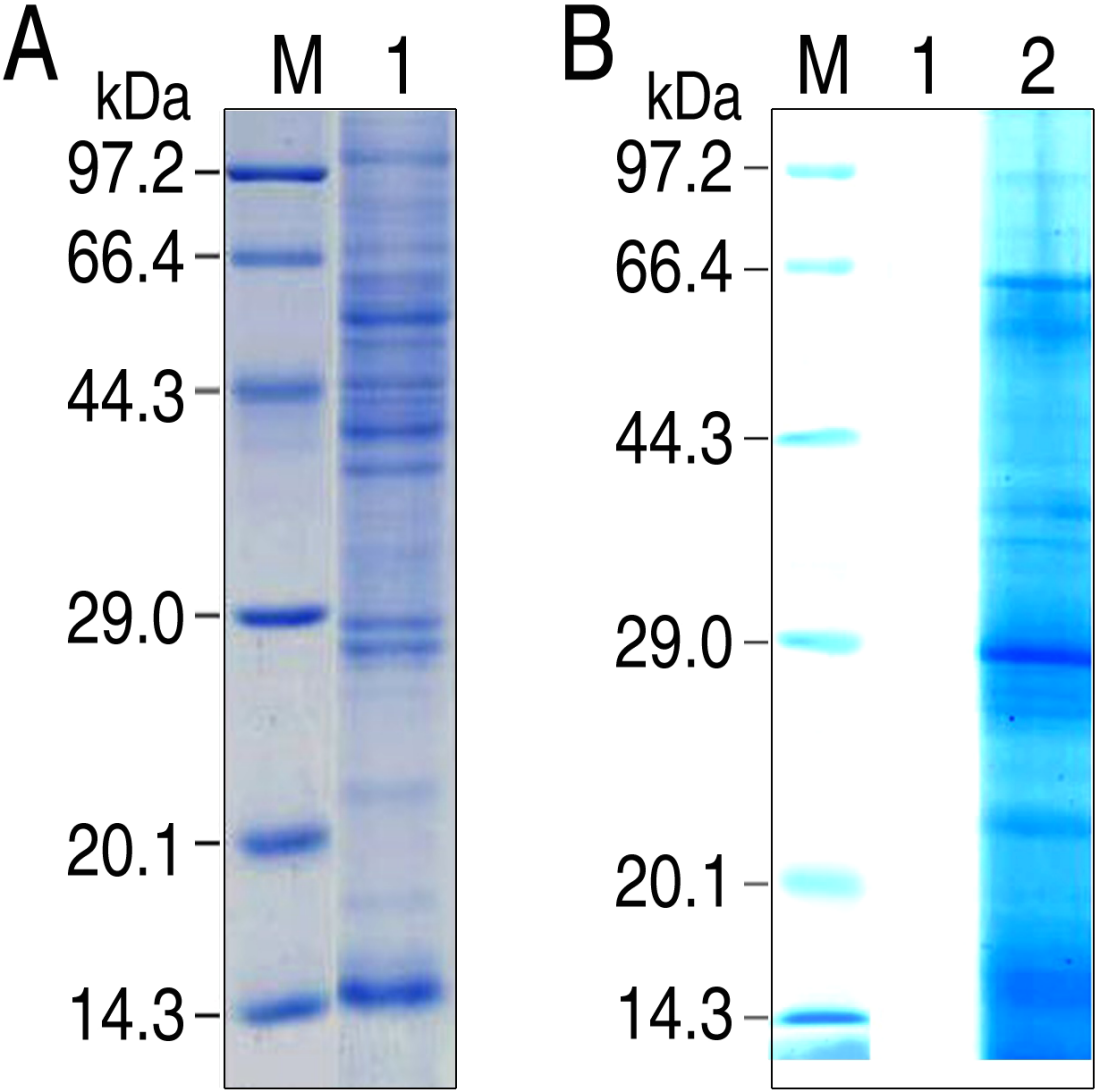
SDS-PAGE analysis for whole proteins **(A)** and poly(I:C)-bound proteins **(B)** of *Danio rerio*. **(A)** M, protein marker; 1, Whole proteins. **(B)** M, protein marker; 1, Control; 2, Proteins binding with poly(I:C).

To identify poly(I:C)-interacting proteins in fish, whole protein extracts were prepared from homogenized zebrafish (**Figure 1A**). Target proteins were captured using resin-immobilized poly(I:C) affinity chromatography. The bound proteins were then separated by SDS-PAGE (**Figure 1B**) and identified by liquid chromatography-tandem mass spectrometry (LC-MS/MS). This approach identified 27 putative binding poly(I:C)-binding proteins (**Table 1**), demonstrating that poly(I:C) can interact with multiple cellular proteins in zebrafish. Functional annotation of the 27 identified proteins revealed that they fall into several broad categories. These include metabolism-related proteins such as apolipoproteins (Apoa1, Apoa2) and ATP synthase subunit Atp5a1; cytoskeletal and contractile proteins represented by actins (Actb1, Actb2), tropomyosin (Tpma), myosin light chain (Mylipa), alpha-actinin (Actn3b), troponin (Tnni2a.3), cofilin (Cfl2l), and keratin (zgc:92533); and immune-related proteins including complement component 1 Q subcomponent-binding protein (C1qbp), lysozyme (Lyz), and F-box only protein 2 (Fbxo2). We also identified RNA-binding and translation-related proteins such as acidic leucine-rich nuclear phosphoprotein 32 family member E (Anp32e) and ribosomal proteins (Rplp2, Rplp2l), as well as oxygen transporters (Hbaa1, Ba1, and the hemoglobin-like protein si:ch211-5k11.8), signal transduction proteins (calmodulin Calm2b, G-protein subunit Gnb1, and VAT-1 homolog Vat1), redox regulators (prolyl 4-hydroxylase P4htm, thioredoxin zgc:56493, and Vat1), and components of the ubiquitin-proteasome system (Skp1, Fbxo2). This functional diversity suggests that poly(I:C) may influence a wide range of cellular processes beyond canonical immune signaling.

**Table 1:** Information of *Danio rerio* proteins which interact with poly(I:C).

Gene	Protein description	Mol. weight [kDa]	Function
*apoa1*	Apolipoprotein A-Ib	30.139	Lipid metabolism and transport
*apoa2*	Apolipoprotein A-II	15.537	Lipid metabolism and transport
*Tpma*	Tropomyosin alpha-1 chain	32.722	Muscle contraction and cytoskeleton
*actn3b*	Alpha-actinin	103.860	Muscle contraction and cytoskeleton
*zgc:92533*	Keratin 23	49.994	Muscle contraction and cytoskeleton
*actb1*	Actin, cytoplasmic 1	41.766	Muscle contraction and cytoskeleton
*actb2*	Actin, cytoplasmic 2	41.752	Muscle contraction and cytoskeleton
*tnni2a.3*	Troponin I, skeletal, fast 2a, tandem duplicate 3	19.733	Muscle contraction and cytoskeleton
*Mylipa*	Myosin light chain, phosphorylatable, fast skeletal muscle b	19.122	Muscle contraction and cytoskeleton
*cfl2l*	Cofilin 2, like	18.770	Muscle contraction and cytoskeleton
*calm2b*	Calmodulin	16.837	Signal transduction
*gnb1*	Guanine nucleotide-binding protein G(I)/G(S)/G(T) subunit beta-1	37.286	Signal transduction
*si:ch211-5k11.8*	Novel protein similar to zebrafish hemoglobin alpha-adult 1	15.565	Oxygen transport and homeostasis
*hbaa1*	Hemoglobin subunit alpha	15.522	Oxygen transport and homeostasis
*ba1*	Hemoglobin subunit beta-1	16.389	Oxygen transport and homeostasis
*c1qbp*	Complement component 1, q subcomponent binding protein	30.029	Immune and defense response
*Lyz*	Lysozyme	17.170	Immune and defense response
*vat1*	Synaptic vesicle membrane protein VAT-1 homolog	53.562	Redox regulation and cellular homeostasis
*p4htm*	Prolyl 4-hydroxylase transmembrane (P4HTM)	57.517	Redox regulation and cellular homeostasis
*zgc:56493*	Thioredoxin	12.014	Redox regulation and cellular homeostasis
*crybb3*	Crystallin, beta B3	28.827	Lens structural component
*anp32e*	Acidic leucine-rich nuclear phosphoprotein 32 family member E (ANP32E)	28.135	Gene expression and translation
*rplp2*	Ribosomal protein, large P2 (RPLP2)	11.712	Gene expression and translation
*rplp2l*	Ribosomal protein, large P2, like	11.638	Gene expression and translation
*skp1*	S-phase kinase-associated protein 1A	18.690	Protein homeostasis and ubiquitination
*fbxo2*	F-box only protein 2 (FBXO2)	20.029	Protein homeostasis and ubiquitination
*atp5a1*	ATP synthase, mitochondrial F1 complex, alpha subunit 1	59.743	Energy metabolism

### 3.2 Bioinformatics analysis of poly(I:C)-binding proteins

**Figure 2:**
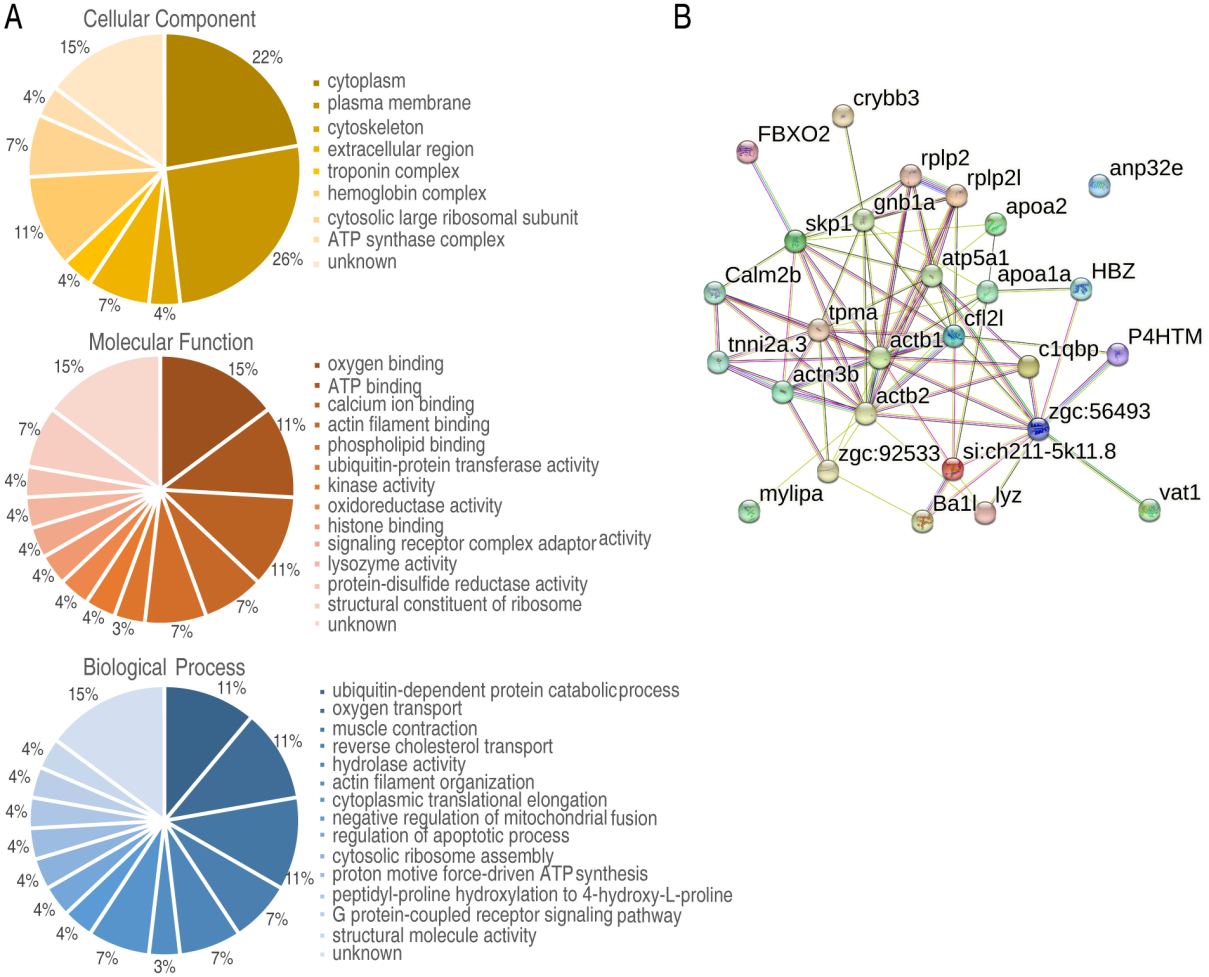
Functional classification and interaction network of poly(I:C)-binding proteins. **(A)** Gene Ontology (GO) classification of the 27 identified proteins. **(B)** Protein-protein interaction (PPI) network constructed using STRING (v11.0) with Markov clustering (MCL) algorithm.

Gene Ontology (GO) enrichment analysis revealed that poly(I:C)-binding proteins are predominantly associated with metabolic/energy-related processes and cytoskeletal organization (**Figure 2A**). Cellular Component analysis showed that most proteins localized to the cytoplasm, plasma membrane, and cytoskeleton, with additional representation in the extracellular region, troponin complex, hemoglobin complex, cytosolic large ribosomal subunit, and ATP synthase complex. Molecular Function analysis identified binding activities (oxygen, ATP, calcium ion, actin filament, and phospholipid binding) as dominant terms, followed by enzymatic functions including ubiquitin-protein transferase, kinase, and oxidoreductase activities. For Biological Process, metabolic processes (ubiquitin-dependent protein catabolic process, oxygen transport, proton motive force-driven ATP synthesis, and cytoplasmic translational elongation) and cytoskeletal processes (muscle contraction, actin filament organization, and regulation of mitochondrial fusion) were the two most prominent groups, with additional involvement in reverse cholesterol transport, regulation of apoptotic process, peptidyl-proline hydroxylation, and G protein-coupled receptor signaling pathway. These patterns suggest that poly(I:C) engagement may broadly reprogram both metabolic activity and cytoskeletal dynamics in host cells.

Protein-protein interaction (PPI) network analysis using STRING (v11.0) with Markov clustering (MCL) revealed that 26 of the 27 identified proteins (excluding anp32e) formed an interconnected network (**Figure 2B**). A dense cytoskeletal core cluster centered on *actb1* (top hub gene), *actb2*, *tpma*, *cfl2l*, and *actn3b* occupied the network center, collectively coordinating actin filament assembly, stabilization, and dynamic remodeling. Closely associated were *tnni2a.3* and *Calm2b*, linking this core to muscle contraction and calcium signaling. Notably, skp1 served as a bridge node connecting the SCF ubiquitin ligase pathway (via FBXO2) to the cytoskeletal core, indicating coupling between protein quality control and structural remodeling. This central hub extended to peripheral modules involving energy metabolism (*atp5a1*, *hbaa1*, *apoa1a/apoa2*), translation (*rplp2*, *rplp2l*), signal transduction (*gnb1a*), and immune-related functions (*zgc:56493*, *c1qbp*, *lyz*). Notably, P4HTM was positioned as a peripheral node connected to *cfl2l* and *zgc:56493*, with no direct linkage to the cytoskeletal core, suggesting it operates through a distinct functional module independent of the central structural hub. Collectively, these findings demonstrate that poly(I:C)-binding proteins are organized around a cytoskeletal-structural hub, supporting the notion that poly(I:C) may modulate cellular structure and coordinate multiple downstream processes.

### 3.3 Validation of poly(I:C)-bound proteins

**Figure 3:**
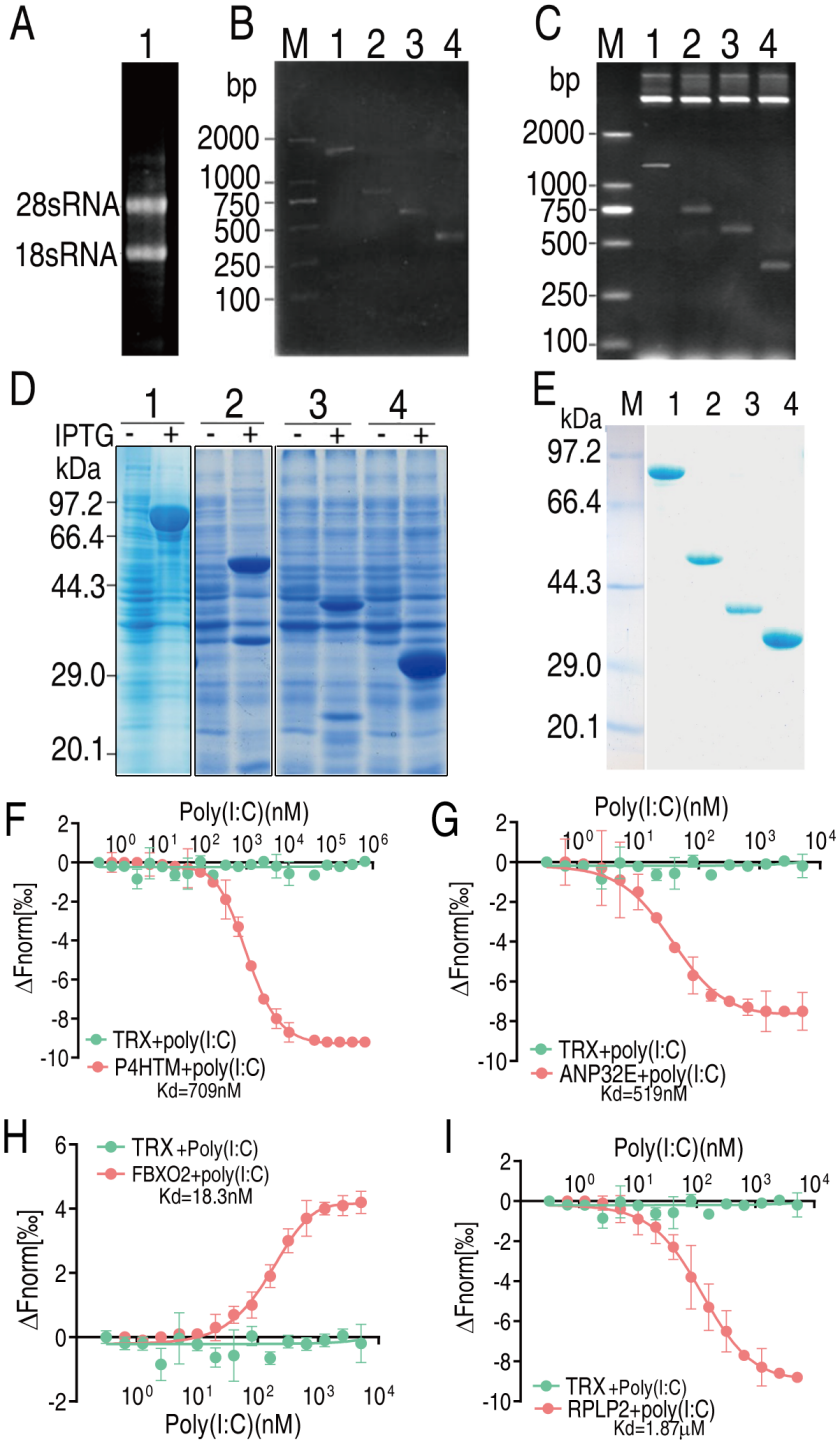
Validation of four poly(I:C)-bound target proteins. **(A)** Integrity of total RNA OD_260_/OD_280_ = 1.86. **(B)** PCR for genes encoding: 1, prolyl 4-hydroxylase (1530bp); 2, Acidic leucine-rich nuclear phosphoprotein 32 family member E (753bp); 3, F-box only protein 2 (534bp); 4, ribosomal protein, large P2 (348bp). **(C)** Double enzymes’ digestion for four plasmids. 1, *p4htm* 1530bp; 2, *anp32e* (753bp) 3, *fbxo2* (534bp); 4, *rplp2* (348bp). M, Marker. **(D)** SDS-PAGE for expression of four *Danio rerio* proteins, the recombinant proteins were expressed with an N-terminal thioredoxin (Trx) tag (approximately 20 kDa). 1, P4HTM (57.5 kDa + 20 kDa); 2, ANP32E (28.1 kDa + 20 kDa); 3, FBXO2 (20 kDa + 20 kDa); 4, RPLP2 (11.7 kDa + 20 kDa). **(E)** Purity of four proteins. 1, P4HTM (57.5 kDa + 20 kDa); 2, ANP32E (28.1 kDa + 20 kDa); 3, FBXO2 (20 kDa + 20 kDa); 4, RPLP2 (11.7 kDa + 20 kDa). **(F-I)** MST for interaction between poly(I:C) and prolyl 4-hydroxylase **(F)**, ANP32E **(G)**, F-box only protein 2 **(H)**, and ribosomal protein, large P2 **(I)** in *Danio rerio.* The purified thioredoxin (Trx) tag protein alone served as a negative control in the MST binding assays.

To validate the reliability of the interactions, four proteins were selected from the 27 identified poly(I:C)-binding candidates following a two-step process. First, the 27 proteins were grouped into eight GO-defined functional categories: (1) cytoskeletal/contractile, (2) metabolic/energy, (3) oxygen transport, (4) immune defense, (5) redox regulation, (6) signal transduction, (7) ubiquitin-proteasome, and (8) gene expression/translation. Second, one representative protein per key functional category was selected to ensure broad coverage of the interactome’s functional breadth: P4HTM (redox regulation/oxygen sensing), ANP32E (gene expression/translation), FBXO2 (protein homeostasis and ubiquitination), and RPLP2 (gene expression and translation/ribosomal machinery). Notably, these four proteins also occupy structurally distinct positions in the PPI network (**Figure 2B**) — FBXO2 bridges the ubiquitin pathway to the cytoskeletal core via skp1; P4HTM is a peripheral node linked only to cfl2l and zgc:56493; ANP32E is the sole completely isolated node; and RPLP2 occupies the peripheral translation module — ensuring the validated set is representative of both the functional and network architecture of the full interactome. The genes encoding these proteins were cloned and expressed in *Escherichia coli* BL21, and the recombinant proteins were purified using nickel affinity chromatography (**Figures 3A–E**). The binding affinity between the recombinant proteins and poly(I:C) was assessed by microscale thermophoresis (MST). The dissociation constants (Kd) for poly(I:C) binding to P4HTM, ANP32E, FBXO2, and RPLP2 were determined to be 0.709 μM, 0.519 μM, 0.0183 μM, and 1.87 μM, respectively (**Figures 3F–I**). In contrast, no specific binding was detected for the thioredoxin (Trx) tag protein alone (vector control), indicating negligible non-specific interaction with poly(I:C) under the same conditions. These results confirm that all four selected proteins specifically interact with poly(I:C).

### 3.4 Functional validation of poly(I:C)-bound proteins

**Figure 4:**
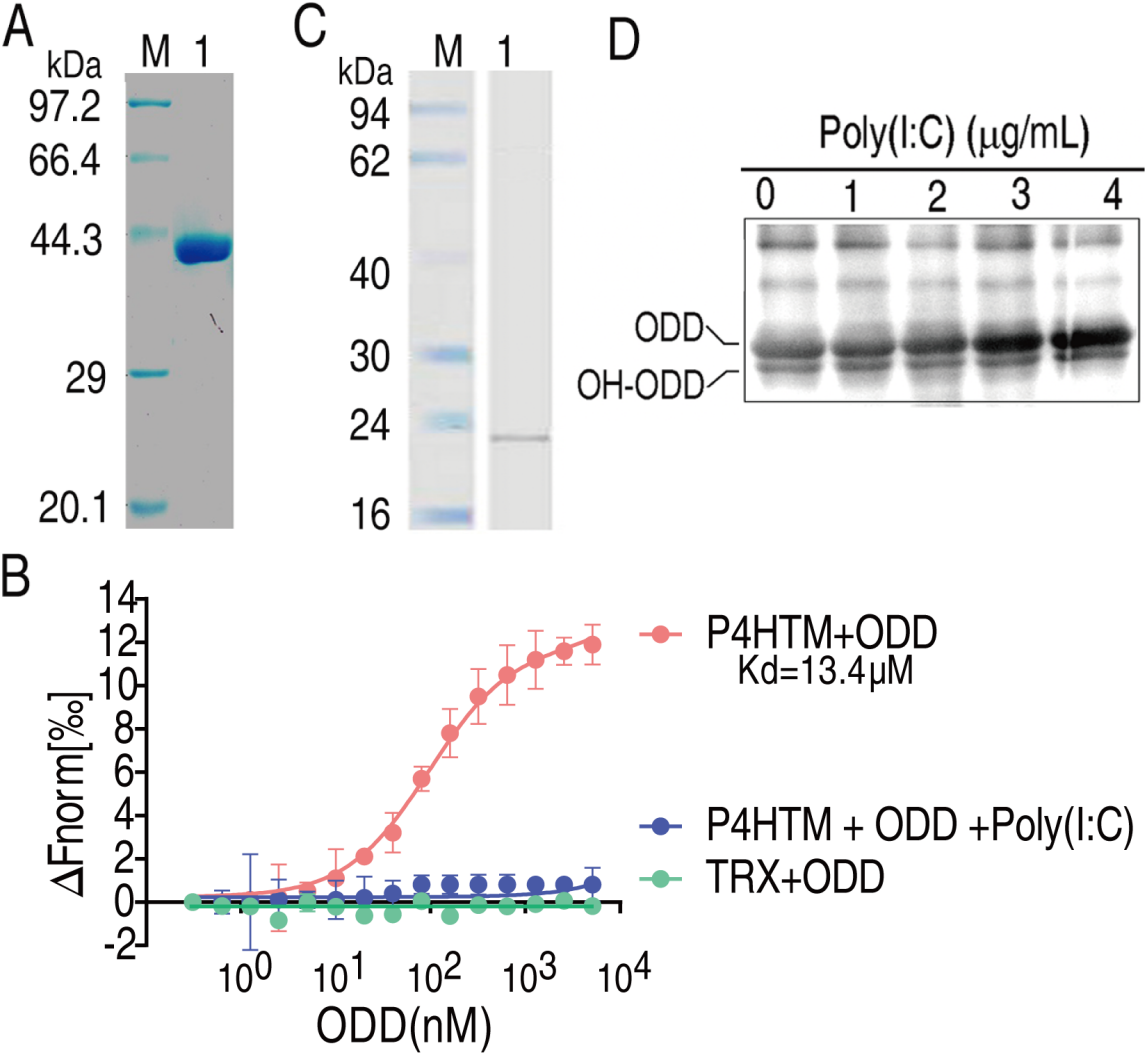
Poly(I:C) impacts the interaction between P4HTM and ODD. **(A)** Purity of *Danio rerio* oxygen-dependent degradation domain recombination protein. M: Marker 1: ODD (21.6 kDa + 20 kDa). **(B)** MST for the binding between P4HTM and ODD in the absence or presence of poly(I:C). **(C)** Verification of antiserum specificity to ODD. **(D)** Western blot for ODD and OH-ODD in the absence or presence of the indicated concentrations of poly(I:C). The upper band represents unmodified ODD, and the lower band corresponds to hydroxylated ODD (OH-ODD). The antibody recognizes both forms, allowing visualization of the poly(I:C) dose-dependent mobility shift.

P4HTM (prolyl 4-hydroxylase) regulates cellular oxygen sensing by hydroxylating two prolyl residues in the oxygen-dependent degradation domain (ODD) of HIF-1α (23). Given that poly(I:C) binds to P4HTM, we hypothesized that this interaction might modulate ODD hydroxylation. To test this, we cloned and expressed the ODD gene, purified the recombinant protein, and performed microscale thermophoresis (MST) analysis (**Figure 4A**). The MST results revealed that the interaction between P4HTM and ODD (Kd = 13.4 μM) was abolished upon poly(I:C) treatment, with no detectable binding signal observed within the tested concentration range (**Figure 4B**). This suggests that poly(I:C) competitively inhibits P4HTM-ODD binding by occupying P4HTM’s interaction site. Consistent with this finding, Western blot analysis demonstrated that poly(I:C) treatment reduced hydroxylated ODD (OH-ODD) levels and induced a dose-dependent retardation in ODD electrophoretic mobility (**Figures 4C, D**). Together, these results indicate that poly(I:C) binding to P4HTM impairs its hydroxylation activity toward ODD, providing a potential mechanism by which poly(I:C) may influence HIF-1α stability and oxygen-sensing pathways.

### 3.5 Response of poly(I:C)-bound proteins to poly(I:C) stimulation

**Figure 5:**
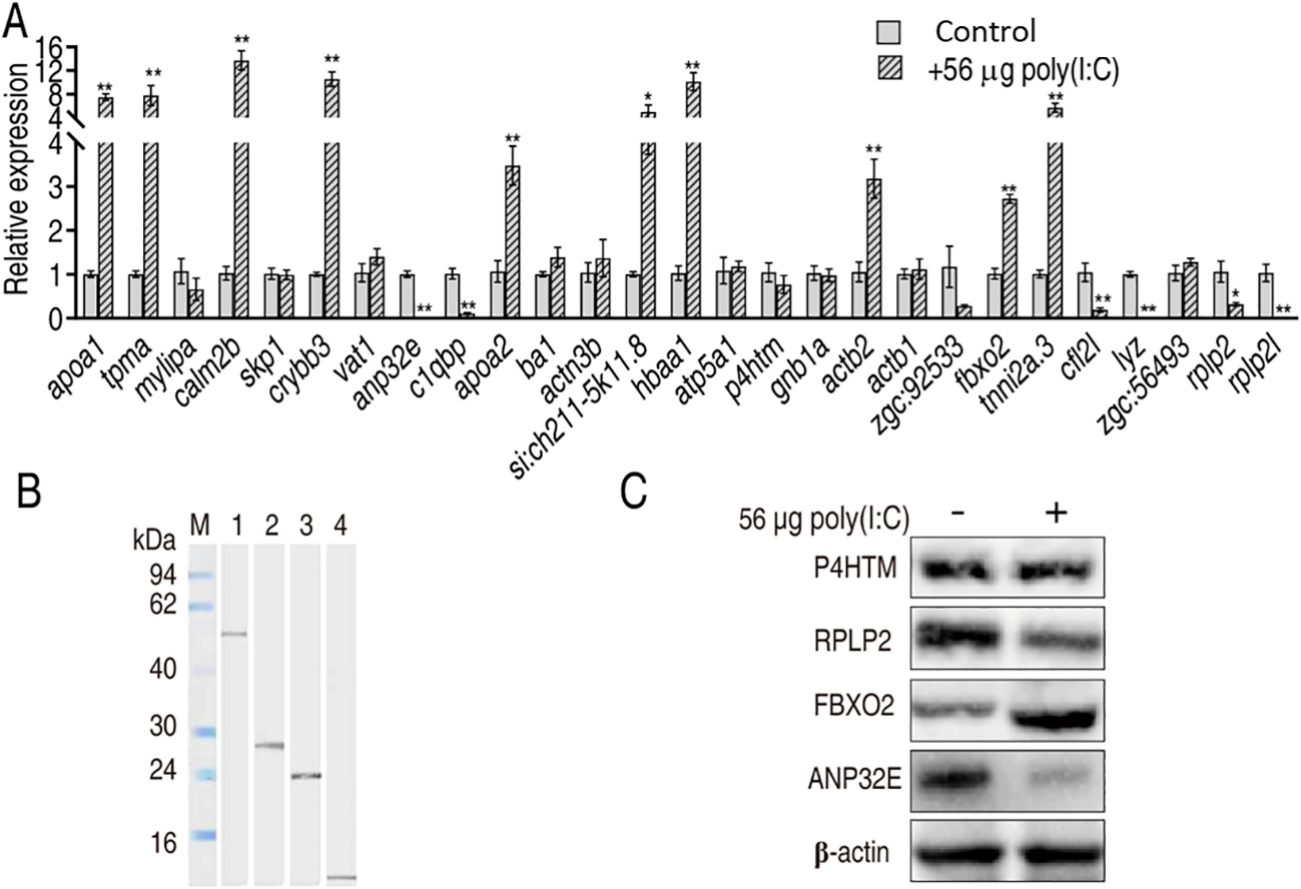
Gene expression and protein abundance of poly(I:C)-binding proteins to poly(I:C) stimulation. **(A)** qRT-PCR for expression of genes encoding 27 poly(I:C)-bound proteins in zebrafish with and without poly(I:C) stimulation. Data are presented as mean ± SD. Statistical significance was determined by Student’s t-test compared to the saline control: *P < 0.05, **P < 0.01. **(B)** Verification of antiserum specificity of proteins. 1, P4HTM (57.5kDa); 2, ANP32E (28.1kDa); 3, FBXO2 (20kDa); 4, RPLP2 (11.7kDa). **(C)** Western blot for abundance of four representative proteins (P4HTM, ANP32E, FBXO2, and RPLP2) in zebrafish with and without poly(I:C) stimulation.

To investigate how poly(I:C)-bound proteins respond to poly(I:C) stimulation, we analyzed both gene expression and protein abundance changes following poly(I:C) challenge. For gene expression analysis, qRT-PCR revealed that among the 27 bound protein genes in zebrafish, 10 (*apoa1, tpma, calm2b, crybb3, apoa2, hbaa1, si:ch211-5k11.8, actb2, fbxo2, tnni2a.3*) were upregulated, 6 (*anp32e, c1qbp, cfl2l, lyz, rplp2, rplp2l*) were downregulated, and 11 (*mylipa, skp1, vat1, zgc:56493, ba1, actn3b, atp5a1, p4htm, gnb1, actb1, zgc:92533*) remained unchanged (**Figure 5A**). For protein analysis, antisera generated against the four purified proteins (P4HTM, ANP32E, FBXO2, and RPLP2) detected single bands at expected molecular weights by Western blot, showing increased FBXO2, decreased RPLP2 and ANP32E, and stable P4HTM levels upon poly(I:C) stimulation (**Figures 5B, C**).

### 3.6 Response of genes encoding poly(I:C)-bound proteins to bacterial infection

**Figure 6:**
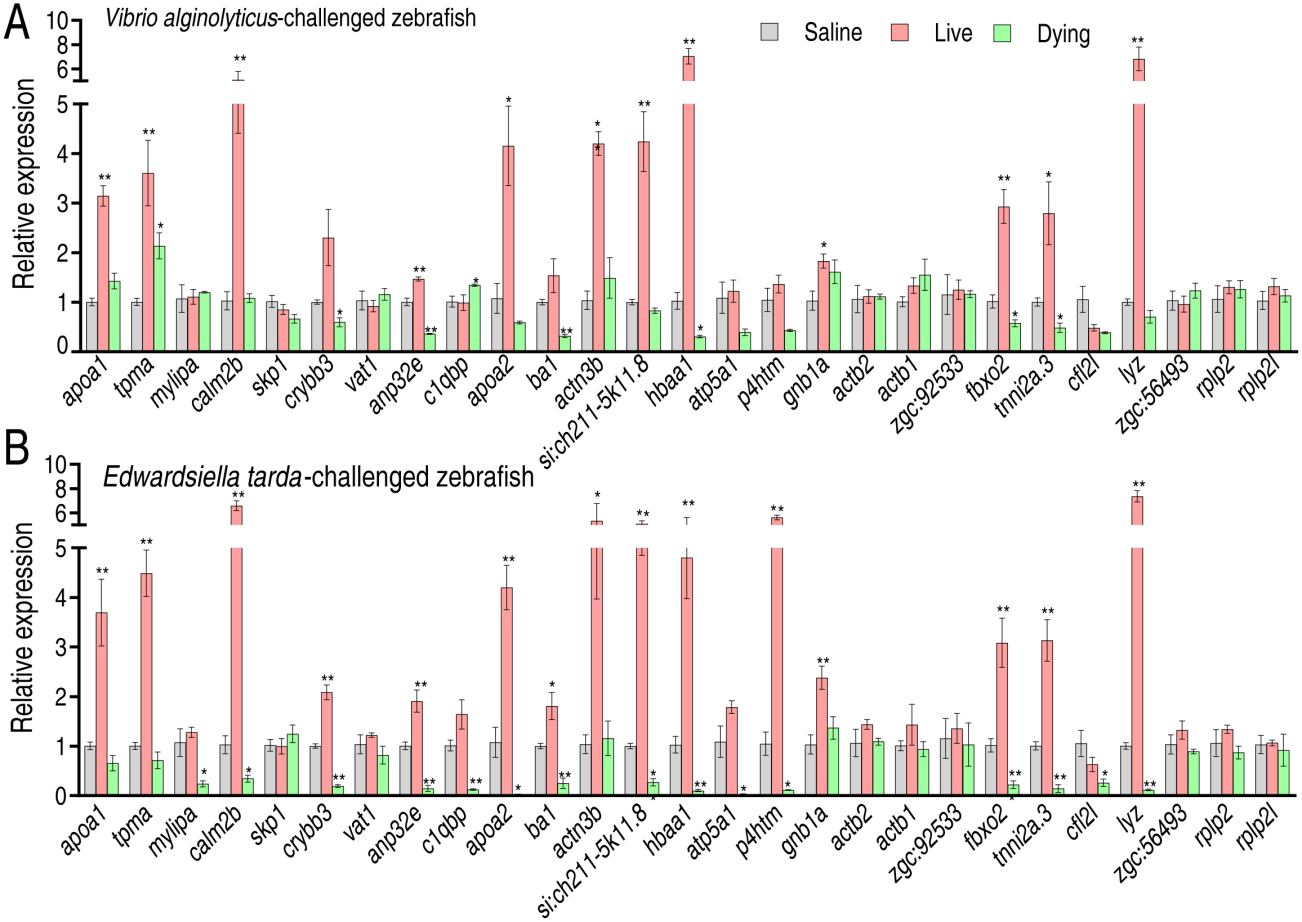
Expression of Poly(I:C) related genes in live and dying zebrafish post infection by *V. alginolyticus* **(A)** and *E. tarda* **(B)**. Gene expression was analyzed by qRT-PCR and normalized to the saline-injected control group (set to 1, indicated by the gray box in the key). Data are presented as mean ± SD. Statistical significance was determined by Student’s t-test compared to the saline control: *P < 0.05, **P < 0.01.

**Figure 7:**
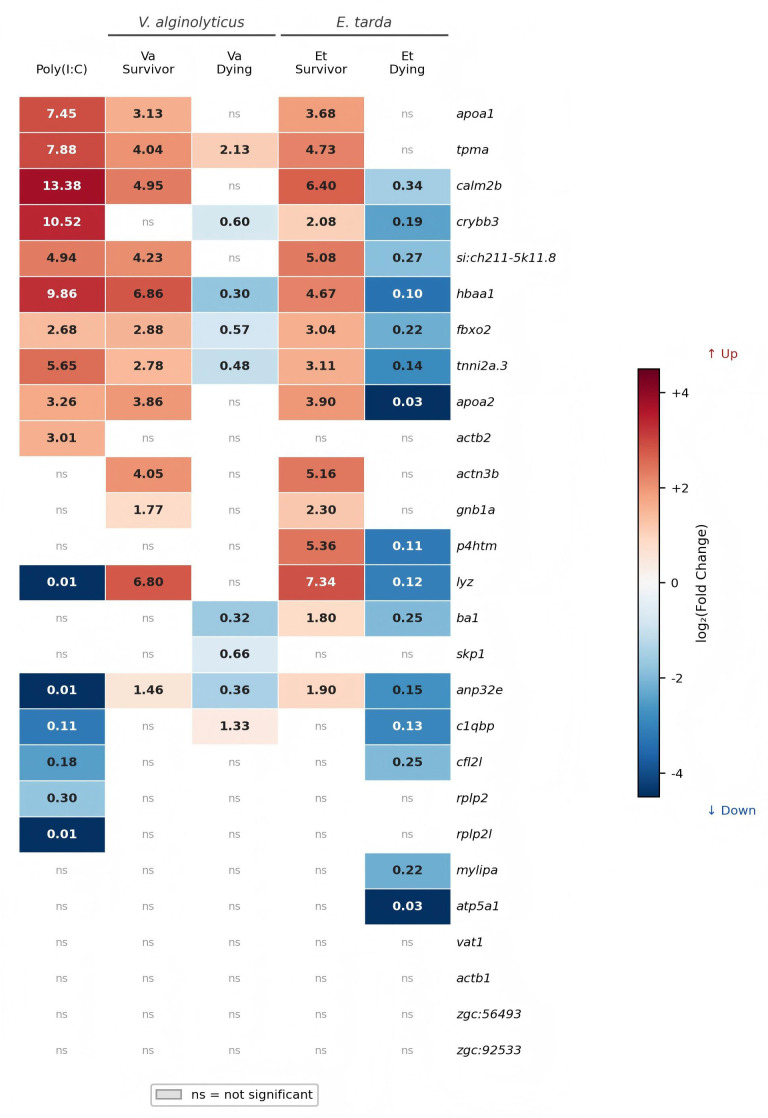
Heatmap comparison of Poly(I:C)-binding protein-encoding gene expression across Poly(I:C) stimulation and bacterial infection outcomes in zebrafish. Gene expression of the 27 poly(I:C)-binding protein-encoding genes was analyzed by qRT-PCR. Data represent fold changes (log_2_) relative to the saline-injected control group (set to 1). Numbers in colored cells indicate fold-change values; cells marked “ns” indicate no statistically significant difference. Statistical significance was determined by Student’s t-test (P < 0.05, P < 0.01). Va, *Vibrio alginolyticus*; Et, *Edwardsiella tarda*; Survivor, fish that survived bacterial infection; Dying, moribund fish collected at 24 h post-infection.

To examine the transcriptional response of genes encoding poly(I:C)-bound proteins under different conditions, we compared gene expression patterns among: (1) poly(I:C)-treated zebrafish (as previously reported), (2) surviving fish post *V. alginolyticus* or *E. tarda* infection, and (3) dying fish (exhibiting abdominal swelling and bradykinesia to EIB202 group, rotate in circles with their belly facing upwards, swollen abdomen and dark body color to Vibrio group) post infection. Among the 27 genes analyzed, distinct expression profiles emerged based on functional categories (**Figures 6**, **7**).

Genes involved in metabolic and structural processes, including those related to lipid transport (*apoa1*, *apoa2*), cytoskeletal organization (*tpma*, *tnni2a.3*), and oxygen transport (*hbaa1*), were generally upregulated in both poly(I:C)-treated fish and infection survivors, but were downregulated or unchanged in dying fish. A second group of genes with stable expression across all conditions included *vat1*, *actb1*, *zgc:92533*, and *zgc:56493*. Genes unaffected by poly(I:C) but responsive to infection, such as those involved in protein turnover (*skp1*) and metabolism (*atp5a1*, *p4htm*), showed infection-induced changes regardless of poly(I:C) pretreatment. In contrast, immune-related genes displayed divergent regulation patterns. For instance, while the lysozyme gene *lyz* was suppressed by poly(I:C) treatment, it was strongly upregulated in infection survivors.

## 4 Discussion

Poly(I:C) is a well-characterized viral mimic known to activate immune responses through TLR-3, RIG-I, and MDA-5 receptors, inducing type I interferon and pro-inflammatory cytokine production (1). Our study significantly expands this understanding by identifying 27 novel poly(I:C)-binding proteins through affinity-proteomics, with four (P4HTM, ANP32E, FBXO2, and RPLP2) rigorously validated by MST. Most remarkably, we discovered a striking correlation in gene expression patterns between poly(I:C)-treated fish (which were not subjected to bacterial infection) and infection survivors that was diametrically opposed to the patterns observed in dying fish. This comparison, made exclusively at the transcriptional level across independent experiments, provides crucial insights into the potential protective mechanism of poly(I:C) against bacterial pathogens.

The 27 poly(I:C)-bound proteins identified in this study represent diverse functional categories with established roles in viral pathogenesis, including structural components (*actn3b/*α-actinin*, actb1/actb2, tpma, mylipa*), metabolic regulators (*atp5a1*/ATP5A1, *p4htm, calm2*/CALM2), immune-related factors (*c1qbp*/C1QBP, *lyz, apoa1*/ApoA1, *apoa2*/ApoA2), ribosomal proteins (*rplp2, rplp2l*), and other regulatory molecules (ANP32E, *cfl2l, ba1, hbaa1*). Strikingly, a literature survey suggests that 24 of these 27 proteins have documented interactions with various viruses: α-actinin facilitates HCV replication (24); ribosomal proteins support dengue and coronavirus infections (25, 26); apolipoproteins modulates dengue virus immune evasion (27); C1QBP interacts with HSV-1 ubiquitin ligase (28); CALM2 is essential for influenza replication (29); GNB1 facilitates influenza virus assembly and release (30); and the actin-myosin network supports viral formation (31). The remaining three proteins (tnni2a.3, zgc:92533, zgc:56493) represent potentially novel viral interaction targets requiring further investigation. This comprehensive profiling reveals that poly(I:C) selectively targets host factors with established roles in viral pathogenesis, suggesting that these interactions may represent evolutionarily conserved viral manipulation sites that poly(I:C) mimics to trigger antiviral responses. It should be noted that this viral association remains an inference based on existing literature rather than direct experimental evidence from our study; nonetheless, it provides valuable context for understanding the potential biological relevance of the identified interactors. Notably, similar divergent transcriptomic responses between resistant/surviving and susceptible/dying fish have been reported in fish viral infection models, including VHSV-challenged rainbow trout (32), SVCV-infected zebrafish (33), and rhabdovirus-infected largemouth bass (34), where surviving fish consistently display stronger induction of innate immune, metabolic, and cytoskeletal gene networks compared to dying fish. This cross-pathogen conservation further supports the notion that the protective gene expression signature identified in our study may represent a broadly conserved teleost host defense strategy.

Extending this parallel, the functional profile of poly(I:C)-binding proteins identified here is consistent with cellular processes altered during fish rhabdovirus infection, further supporting poly(I:C)’s role as a viral mimic. Rhabdovirus (VHSV, HIRRV) infection in fish induces cytoskeletal reorganization involving actin, tropomyosin, and α-actinin (35); disruption of lipid and energy metabolism, and impaired cellular redox status (36); and apoptosis via caspase activation in fish cells (37). These processes directly correspond to the functional categories of poly(I:C)-binding proteins identified in our study: cytoskeletal components (Actb1/2, Tpma, Actn3b, Cfl2l), lipid/energy metabolism regulators (Apoa1/2, Atp5a1), redox-related proteins (thioredoxin, P4HTM, VAT-1), and apoptosis/protein turnover mediators (FBXO2, C1QBP). This convergence reinforces the notion that poly(I:C) targets evolutionarily conserved host factors exploited by rhabdoviruses.

Our validation experiments confirmed specific poly(I:C) binding to P4HTM, ANP32E, FBXO2, and RPLP2. The functional impact was demonstrated through P4HTM’s inhibited hydroxylation of ODD upon poly(I:C) binding. P4HTM is a transmembrane prolyl hydroxylase involved in oxygen sensing and cellular adaptation to hypoxic stress. Its identification in our pulldown, despite not being a direct nucleic acid sensor, suggests potential crosstalk between hypoxic stress pathways and innate immune signaling following poly(I:C) treatment. The absence of TLR3, RIG-I, and MDA5 among the identified proteins likely reflects the intrinsic dynamic range limitation of mass spectrometry. This technique is inherently biased toward high-abundance ‘proteomic sponge’ proteins (e.g., actin, metabolic enzymes), which can suppress signals from low-copy-number signaling receptors. Thus, this technical constraint—not subcellular localization—accounts for their non-detection. Notably, P4HTM showed no significant change in either mRNA expression or protein abundance upon poly(I:C) stimulation (Section 3.5), consistent with a mechanism of functional modulation rather than abundance regulation — poly(I:C) inhibits P4HTM’s enzymatic activity toward ODD without altering its expression levels.

The inhibition of P4HTM by poly(I:C) may reflect a conserved viral strategy to stabilize HIF-1α, which confers multiple advantages for virus survival and replication. First, HIF-1α stabilization drives a metabolic shift from oxidative phosphorylation to glycolysis, providing the rapid ATP production and biosynthetic intermediates required for viral replication — a mechanism demonstrated for H1N1 influenza and RSV (38, 39). Second, HIF-1α stabilization can suppress early apoptosis in infected cells, prolonging host cell survival and creating a permissive environment for viral propagation (40). Third, in fish virology specifically, ISKNV has been shown to exploit the HIF-1α pathway via a positive feedback mechanism to enhance replication, directly supporting the relevance of this axis in aquatic virus–host interactions (41). Taken together, poly(I:C)-mediated inhibition of P4HTM activity, and consequent HIF-1α stabilization, may recapitulate an evolutionarily conserved viral immune evasion and metabolic reprogramming strategy.

Beyond the P4HTM–ODD axis, the GO enrichment and PPI network data together suggest that poly(I:C) may influence a wide range of cellular processes beyond canonical immune signaling. The predominance of cytoskeletal, metabolic, and structural proteins among the identified interactors indicates that poly(I:C) engagement may broadly reprogram both metabolic activity and cytoskeletal dynamics in host cells. PPI network analysis further reveals that these binding proteins are organized around a cytoskeletal-structural hub, supporting the notion that poly(I:C) may modulate cellular architecture and coordinate multiple downstream processes in a network-dependent manner. Future studies employing cytoskeletal perturbation assays and metabolic flux analyses will be required to directly test these mechanistic inferences.

The most significant finding emerged from transcriptional analysis, which revealed that 9 specific genes (*apoa1, tpma, calm2b, crybb3, apoa2, si:ch211-5k11.8, hbaa1, fbxo2, tnni2a.3*) exhibited identical upregulation patterns in both poly(I:C)-treated fish and infection survivors, while showing opposite (downregulated or unchanged) expression in dying fish. This remarkable correlation suggests that these genes may constitute a candidate protective signature whose expression is associated with survival outcomes during bacterial infection, though functional validation through gene knockdown or overexpression experiments will be required to establish causality. These genes may serve as both biomarkers for infection resistance and mediators of poly(I:C)’s protective effects against *V. alginolyticus* and *E. tarda* infections. A review of published transcriptomic and proteomic studies on zebrafish and fish responses to rhabdovirus and nodavirus infections reveals that at least four of these nine genes have previously been implicated in viral infections: *apoa1* upregulation is associated with survival in VHSV-infected zebrafish and ApoA1 protein exhibits direct antiviral activity against HSV (32, 42); fbxo2 is activated by EBV to restrict viral infectivity via proteasomal degradation of viral glycoprotein B (43), and the closely related *fbxo3* regulates antiviral responses during SVCV infection in zebrafish (44); *hbaa1*-related hemoglobin proteins have been identified as survival-associated biomarkers in VHSV-infected zebrafish (33); and *tpma*-associated cytoskeletal reorganization has been documented in rhabdovirus-infected fish (34). The remaining five genes (*calm2b, crybb3, apoa2, si:ch211-5k11.8, tnni2a.3*) have not yet been explicitly reported in the context of fish viral immunity, representing important targets for future investigation. This cross-pathogen conservation strengthens the proposition that these genes represent broadly relevant host defense mediators beyond bacterial infection alone. At the LD50 challenge dose, the infected population naturally segregated into surviving (~50%) and dying (~50%) individuals at 24 h post-infection, effectively representing resistant and susceptible subpopulations, respectively. However, poly(I:C) pretreatment induces an expression profile that shares similarities with that observed in infection survivors for a subset of genes involved in metabolism and structure, although notable exceptions such as *lyz* indicate that the protective effect may involve complex, gene-specific regulatory mechanisms rather than simply mimicking the survivor state. This suggests that poly(I:C) pretreatment may help establish a protective transcriptional environment that enhances host resistance against subsequent bacterial challenges.

While our findings provide novel insights into poly(I:C)’s binding partners and protective mechanisms, certain limitations should be considered when interpreting these results. First, although a bead-only control was not included in the pull-down experiment, the core findings are supported by independent validation approaches. Specifically, the binding interactions of four selected proteins (P4HTM, ANP32E, FBXO2, and RPLP2) identified via pull-down were independently confirmed by microscale thermophoresis (MST) with quantifiable dissociation constants, while no binding was detected for the tag-only control (Section 3.3). Their functional relevance was further supported by Western blot analysis showing protein abundance changes upon poly(I:C) stimulation (Section 3.5), functional inhibition assays (Section 3.4), and qRT-PCR-based gene expression profiling. Thus, while we acknowledge this technical limitation, the convergence of multiple lines of evidence strongly supports the validity of our major conclusions. Second, the primary constraint of this study is the lack of functional experiments, such as gene knockdown or overexpression, to validate the biological roles of the identified poly I:C-binding proteins. While our proteomic screening provides a valuable list of candidate interactors, the causal relationship between these proteins and poly I:C-mediated immune enhancement in fish remains correlational. Future work incorporating functional assays will be necessary to confirm the necessity and sufficiency of these candidates in mediating the observed effects. Third, the affinity proteomics was performed using naïve (unstimulated) zebrafish. Pre-stimulation with poly(I:C), LPS, bacteria, or virus could expand the identified interactome by revealing inducible binding partners; future studies comparing naïve and stimulated fish proteomes under identical affinity-pulldown conditions would provide a more comprehensive interaction map.

## 5 Conclusion

Our study makes three major contributions: (1) identification of 27 novel poly(I:C)-binding proteins expanding its known interactome; (2) demonstration of functional consequences through P4HTM-ODD interaction inhibition; and most importantly (3) discovery of a conserved gene expression signature (9 genes showing identical upregulation in poly(I:C)-treated and surviving fish but opposite patterns in dying fish) that provides mechanistic insights into the molecular basis underlying the protective effect of poly(I:C) against bacterial infection established in previous work. These findings not only advance our understanding of poly(I:C)’s immunomodulatory mechanisms but also identify potential biomarkers for monitoring infection resistance in fish, with possible implications for developing preventive strategies against bacterial pathogens in aquaculture, and broader relevance to understanding conserved dsRNA–host interactions in vertebrate innate immunity.

## Data Availability

The data underlying this article are available in the Figshare Digital Repository at https://dx.doi.org/10.6084/m9.figshare.29664869.

## References

[1] De Waele J Verhezen T van der Heijden S Berneman ZN Peeters M Lardon F . A systematic review on poly(I:C) and poly-ICLC in glioblastoma: adjuvants coordinating the unlocking of immunotherapy. J Exp Clin Cancer Res. (2021) 40:213. doi: 10.1186/s13046-021-02017-2 34172082 PMC8229304

[2] Golshani M Amani M Amirzadeh F Nazeri E Davar Siadat S Nejati-Moheimani M . Evaluation of poly(I:C) and combination of CpG ODN plus Montanide ISA adjuvants to enhance the efficacy of outer membrane vesicles as an acellular vaccine against Brucella melitensis infection in mice. Int Immunopharmacol. (2020) 84:106573. doi: 10.1016/j.intimp.2020.106573 32454410

[3] Souza-Moreira L Tan Y Wang Y Wang JP Salkhordeh M Virgo J . Poly(I:C) enhances mesenchymal stem cell control of myeloid cells from COVID-19 patients. iScience. (2022) 25:104188. doi: 10.1016/j.isci.2022.104188 35402859 PMC8975597

[4] Bianchi F Pretto S Tagliabue E Balsari A Sfondrini L . Exploiting poly(I:C) to induce cancer cell apoptosis. Cancer Biol Ther. (2017) 18:747–56. doi: 10.1080/15384047.2017.1373220 28881163 PMC5678690

[5] Khan ZA Sumsuzzman DM Choi J Kamenos G Hong Y . Pre- and post-conditioning with poly I:C exerts neuroprotective effect against cerebral ischemia injury in animal models: a systematic review and meta-analysis. CNS Neurosci Ther. (2022) 28:1168–82. doi: 10.1111/cns.13851 35510663 PMC9253751

[6] Zhao X Ai M Guo Y Zhou X Wang L Li X . Poly I:C-induced tumor cell apoptosis mediated by pattern-recognition receptors. Cancer Biother Radiopharm. (2012) 27:530–4. doi: 10.1089/cbr.2012.1226 23062195

[7] Kawai T Akira S . Toll-like receptor and RIG-I-like receptor signaling. Ann N Y Acad Sci. (2008) 1143:1–20. doi: 10.1196/annals.1443.020 19076341

[8] Kawai T Akira S . Signaling to NF-κB by toll-like receptors. Trends Mol Med. (2007) 13:460–9. doi: 10.1016/j.molmed.2007.09.002 18029230

[9] Wang YH Zhang YG . Poly (I:C) alleviates obesity related pro-inflammatory status and promotes glucose homeostasis. Cytokine. (2017) 99:225–32. doi: 10.1016/j.cyto.2017.07.011 28757363

[10] Guo C Ye JZ Song M Peng XX Li H . Poly I:C promotes malate to enhance innate immune response against bacterial infection. Fish Shellfish Immunol. (2022) 131:172–80. doi: 10.1016/j.fsi.2022.09.064 36210004

[11] York AG Williams KJ Argus JP Zhou QD Brar G Vergnes L . Limiting cholesterol biosynthetic flux spontaneously engages type I IFN signaling. Cell. (2015) 163:1716–29. doi: 10.1016/j.cell.2015.11.045 26686653 PMC4783382

[12] Zhou ZX Zhang BC Sun L . Poly(I:C) induces antiviral immune responses in Japanese flounder (Paralichthys olivaceus) that require TLR3 and MDA5 and is negatively regulated by Myd88. PloS One. (2014) 9:e112918. doi: 10.1371/journal.pone.0112918 25393122 PMC4231074

[13] Hou Q Gong R Liu X Mao H Xu X Liu D . Poly I:C facilitates the phosphorylation of Ctenopharyngodon idellus type I IFN receptor subunits and JAK kinase. Fish Shellfish Immunol. (2017) 60:13–20. doi: 10.1016/j.fsi.2016.10.042 27815207

[14] Green TJ Chataway T Melwani AR Raftos DA . Proteomic analysis of hemolymph from poly(I:C)-stimulated Crassostrea gigas. Fish Shellfish Immunol. (2016) 48:39–42. doi: 10.1016/j.fsi.2015.11.018 26578249

[15] Zhang J Sun S Mao Y Qiao G Li Q . Identification and analysis of differentially expressed microRNAs in gibel carp Carassius auratus gibelio responding to polyinosinic-polycytidylic acid (poly I:C) stimulation. Fish Shellfish Immunol Rep. (2023) 4:100083. doi: 10.1016/j.fsirep.2023.100083 36660301 PMC9842694

[16] Zhao XL Chen ZG Yang TC Jiang M Wang J Cheng ZX . Glutamine promotes antibiotic uptake to kill multidrug-resistant uropathogenic bacteria. Sci Transl Med. (2021) 13:eabj0716. doi: 10.1126/scitranslmed.abj0716 34936385

[17] Szklarczyk D Gable AL Lyon D Junge A Wyder S Huerta-Cepas J . STRING v11: protein–protein association networks with increased coverage supporting functional discovery in genome-wide experimental datasets. Nucleic Acids Res. (2019) 47:D607–13. doi: 10.1093/nar/gky1131 30476243 PMC6323986

[18] Li H Huang X Zeng Z Peng XX Peng B . Identification of the interactome between fish plasma proteins and Edwardsiella tarda reveals tissue-specific strategies against bacterial infection. Int J Biochem Cell Biol. (2016) 78:260–7. doi: 10.1016/j.biocel.2016.07.021 27458055

[19] Cheng ZX Guo C Chen ZG Yang TC Zhang JY Wang J . Glycine, serine and threonine metabolism confounds efficacy of complement-mediated killing. Nat Commun. (2019) 10:3325. doi: 10.1038/s41467-019-11129-5 31346171 PMC6658569

[20] Jiang M Chen ZG Li H Zhang TT Yang MJ Peng XX . Succinate and inosine coordinate innate immune response to bacterial infection. PloS Pathog. (2022) 18:e1010796. doi: 10.1371/journal.ppat.1010796 36026499 PMC9455851

[21] Zhang DF Ye JZ Dai HH Lin XM Li H Peng XX . Identification of ethanol tolerant outer membrane proteome reveals OmpC-dependent mechanism in a manner of EnvZ/OmpR regulation in Escherichia coli. J Proteomics. (2018) 179:92–9. doi: 10.1016/j.jprot.2018.03.005 29518576

[22] Jiang M Su YB Ye JZ Li H Kuang SF Wu JH . Ampicillin-controlled glucose metabolism manipulates the transition from tolerance to resistance in bacteria. Sci Adv. (2023) 9:eade8582. doi: 10.1126/sciadv.ade8582 36888710 PMC9995076

[23] Koivunen P Tiainen P Hyvärinen J Williams KE Sormunen R Klaus SJ . An endoplasmic reticulum transmembrane prolyl 4-hydroxylase is induced by hypoxia and acts on hypoxia-inducible factor α. J Biol Chem. (2007) 282:30544–52. doi: 10.1074/jbc.M704988200 17726031

[24] Lan S Wang H Jiang H Mao H Liu X Zhang X . Direct interaction between alpha-actinin and hepatitis C virus NS5B. FEBS Lett. (2003) 554:289–94. doi: 10.1016/s0014-5793(03)01163-3 14623081

[25] Campos RK Wong B Xie X Lu YF Shi PY Pompon J . RPLP1 and RPLP2 are essential Flavivirus host factors that promote early viral protein accumulation. J Virol. (2017) 91:e01706-16. doi: 10.1128/JVI.01706-16 27974556 PMC5286887

[26] Dong HJ Wang J Zhang XZ Li CC Liu JF Wang XJ . Proteomic screening identifies RPLp2 as a specific regulator for the translation of coronavirus. Int J Biol Macromol. (2023) 230:123191. doi: 10.1016/j.ijbiomac.2023.123191 36632964 PMC9827737

[27] Coelho DR Carneiro PH Mendes-Monteiro L Conde JN Andrade I Cao T . Apoa1 neutralizes proinflammatory effects of dengue virus NS1 protein and modulates viral immune evasion. J Virol. (2021) 95:e0197420. doi: 10.1128/JVI.01974-20 33827950 PMC8437349

[28] Hou F Sun Z Deng Y Chen S Yang X Ji F . Interactome and ubiquitinome analyses identify functional targets of herpes simplex virus 1 infected cell protein 0. Front Microbiol. (2022) 13:856471. doi: 10.3389/fmicb.2022.856471 35516420 PMC9062659

[29] Bakre A Andersen LE Meliopoulos V Coleman K Yan X Brooks P . Identification of host kinase genes required for influenza virus replication and the regulatory role of microRNAs. PloS One. (2013) 8:e66796. doi: 10.1371/journal.pone.0066796 23805279 PMC3689682

[30] Li F Liu J Yang J Sun H Jiang Z Wang C . H9N2 virus-derived M1 protein promotes H5N6 virus release in mammalian cells: mechanism of avian influenza virus inter-species infection in humans. PloS Pathog. (2021) 17:e1010098. doi: 10.1371/journal.ppat.1010098 34860863 PMC8641880

[31] Kumakura M Kawaguchi A Nagata K . Actin-myosin network is required for proper assembly of influenza virus particles. Virology. (2015) 476:141–50. doi: 10.1016/j.virol.2014.12.016 25543965

[32] Verrier ER Langevin C Benmansour A Boudinot P . Early antiviral response and virus-induced genes in fish. Dev Comp Immunol. (2011) 35:1204–14. doi: 10.1016/j.dci.2011.03.012 21414349

[33] Wang Z Zhu C Sun X Deng H Liu W Jia S . Spring viremia of carp virus infection induces hypoxia response in zebrafish by stabilizing hif1α. J Virol. (2025) 99:e01491-24. doi: 10.1128/jvi.01491-24 39601573 PMC11784138

[34] Fei H Yi SF Zhang HM Cheng Y Zhang YQ Yu X . Transcriptome and 16S rRNA analysis revealed the response of largemouth bass (Micropterus salmoides) to rhabdovirus infection. Front Immunol. (2022) 13:973422. doi: 10.3389/fimmu.2022.973422 36275642 PMC9585208

[35] Encinas P Rodriguez-Milla MA Novoa B Estepa A Figueras A Coll J . Zebrafish fin immune responses during high mortality infections with viral haemorrhagic septicemia rhabdovirus. A proteomic and transcriptomic approach. BMC Genomics. (2010) 11:518. doi: 10.1186/1471-2164-11-518 20875106 PMC2997011

[36] Gu B Pan F Wang H Zou Z Song J Xing J . Untargeted LC-MS metabolomics reveals the metabolic responses in olive flounder subjected to hirame rhabdovirus infection. Front Immunol. (2023) 14:1148740. doi: 10.3389/fimmu.2023.1148740 37711614 PMC10498126

[37] Miest JJ Adamek M Pionnier N Harris S Houston RD Falco A . Differential effects of alloherpesvirus CyHV-3 and rhabdovirus SVCV on apoptosis in fish cells. Vet Microbiol. (2015) 176:19–31. doi: 10.1016/j.vetmic.2014.12.023 25596969

[38] Xu Y Zhong Z Zhang J Cheng M Guo Z Ye F . HIF-1α promotes virus replication and cytokine storm in H1N1 virus-induced severe pneumonia through cellular metabolic reprogramming. Virol Sin. (2024) 39:142–54. doi: 10.1016/j.virs.2023.11.010 38042371 PMC10877445

[39] Chen LF Cai JX Zhang JJ Tang YJ Chen JY Xiong S . Respiratory syncytial virus co-opts hypoxia-inducible factor-1α-mediated glycolysis to favor the production of infectious virus. mBio. (2023) 14:e02110-23. doi: 10.1128/mbio.02110-23 37796013 PMC10653832

[40] Reyes A Corrales N Gálvez NMS Bueno SM Kalergis AM González PA . Contribution of hypoxia inducible factor-1 during viral infections. Virulence. (2020) 11:1482–500. doi: 10.1080/21505594.2020.1836904 33135539 PMC7605355

[41] He J Yu Y Liu W Li Z Qi Z Weng S . Molecular mechanism of infectious spleen and kidney necrosis virus in manipulating the hypoxia-inducible factor pathway to augment virus replication. Virulence. (2024) 15:2349027. doi: 10.1080/21505594.2024.2349027 38680083 PMC11085990

[42] Srinivas RV Venkatachalapathi YV Rui Z Owens RJ Gupta KB Srinivas SK . Antiviral effects of apolipoprotein A-I and its synthetic amphipathic peptide analogs. Virology. (1990) 176:48–57. doi: 10.1016/0042-6822(90)90229-k 2158697

[43] Zhang HJ Tian J Qi XK Xiang T He GP Zhang H . Epstein-Barr virus activates F-box protein FBXO2 to limit viral infectivity by targeting glycoprotein B for degradation. PLoS Pathog. (2018) 14:e1007208. doi: 10.1371/journal.ppat.1007208 30052682 PMC6082576

[44] Li M Gong J Wu S Zhong K Zhang J Guo M . Zebrafish F-box Protein fbxo3 Negatively Regulates Antiviral Response through Promoting K27-Linked Polyubiquitination of the Transcription Factors irf3 and irf7. J Immunol. (2020) 205:1897–908. doi: 10.4049/jimmunol.2000305 32859728

