# Supplementary material for: Identification of poly(I:C) interacted proteins and their regulation in transcription level against bacterial infection in zebrafish
Source: Front Immunol. 2026 Jul 3;17:1848091. doi: 10.3389/fimmu.2026.1848091 (PMC13375457; doi:10.3389/fimmu.2026.1848091)
Supplement: Supplementary file 1 [file Table1.docx]

**Identification of poly(I:C) interacted proteins and their regulation in transcription level against bacterial infection in zebrafish**

Hui-yin Lin, Jiao Xiang, Yi Han, Xuan-xian Peng, Hui Li, Xian-jie Liu

**Supplementary Table 1. Gene clone information of *Danio rerio***

| **Gene** | **Primer sequence (5'to3')** | **Vector** | **Res.site** |
| --- | --- | --- | --- |
| *anp32e* | GGGGGATCCATGGAGATGAAGAAGAGGATT | pET-32a | *Bam*HI |
|  | CCCAAGCTTTTAGTCGTCGTCTTCATCGTC |  | *Hin*dIII |
| *fbxo2* | GGGGGATCCATGCCTGGAAACCTTATAA | pET-32a | *Bam*HI |
|  | CCCAAGCTTTTAGACGTCTACGGTAACAGAG |  | *Hin*dIII |
| *rplp2* | GGGGAATTCATGCGTTACGTGGCCGCTTA | pET-32a | *Eco*RI |
|  | CCCAAGCTTTTAATCAAAGAGGCCAAAGCCC |  | *Hin*dIII |
| *p4htm* | GGGGAATTCATGTTGTCTGGCAAAATGATG | pET-32a | *Eco*RI |
|  | CCCAAGCTTTTACAGGTCTTGGTGAGCTTC |  | *Hin*dIII |
| *odd* | GGGGTCGACGCACCTGCAGCAGGCGACGCCATTAT | pET-32a | *Sal*I |
|  | CCCAAGCTTCTGCTTCACTGTGCTGCTGGTAG |  | *Hin*dIII |

**Supplementary Table 2. Primers for qRT-PCR**

| Gene | Primer | Sequence (5'-3') | Gene | Primer | Sequence (5'-3') |
| --- | --- | --- | --- | --- | --- |
| *apoa1a* | Forward | CAGGCCAATTTGTTCCAGGC | *si:ch211-5k11.8* | Forward | CCCCTGAAGTTCACGTGTCA |
|  | Reverse | TTCCGTCCAGGTTGTCAAGG |  | Reverse | CCGCACAGTGTTGTTGTCAG |
| *tpma* | Forward | AAGGAGAACGCCTTGGACAG | *p4htm* | Forward | TATCAGGGTCAGGGAGCACA |
|  | Reverse | CAACTCATCCTCGGTTGCCT |  | Reverse | AGCGGCTCACTTAACTCCAC |
| *mylipa* | Forward | AAGGCGAACGGAGAAGACTG | *gnb1* | Forward | CACACCGAACGCACAAGAAG |
|  | Reverse | CAGGGCGTGAGATTGTCCAT |  | Reverse | AAATAACGCGAGCTTCACGC |
| *calm2b* | Forward | CATTAGTGCGGCTGAACTGC | *actb2* | Forward | ACTTTGAGCTCCTCCACACG |
|  | Reverse | TGACCTGACCGTCTCCATCA |  | Reverse | CAACCAGTGCGGCAATTTCA |
| *skp1* | Forward | AACCCCCGAGGAGATCAGAA | *actb1* | Forward | TGCTCTGTATGGCGCATTGA |
|  | Reverse | TGCGTCGGGATCTTCACTTC |  | Reverse | GAGGGCAAAGTGGTAAACGC |
| *crybb3* | Forward | ATCAGACCCCTACGTGTGGA | *zgc:92533* | Forward | GCACTGGTTCCAGTCCAAGA |
|  | Reverse | GTTGACAGCTTTCGCACTGG |  | Reverse | TGTGCGCTTAAGCTCTGTGA |
| *vat1* | Forward | GGTTTACCACGCGATCATGC | *fbxo2* | Forward | GACTTGTTGGCGGAGGGTTA |
|  | Reverse | CTGTGAGGGGGTGAGACCTA |  | Reverse | TGCATCCACAGTCTGTTCGG |
| *anp32e* | Forward | AACCTATGAGCAGTTCGCCC | *tnni2a.3* | Forward | GCTGGTATGGGTGGCAGAAA |
|  | Reverse | AAAGGTACTCAAGGGTGCCG |  | Reverse | GTGAATGCGCAGCTCTGTAG |
| *c1qbp* | Forward | ATCAGCCGCTCATTCTGGAG | *cfl2l* | Forward | GCGACGACAAGAAGCACATC |
|  | Reverse | TGCGAAGGCTTTATCACCGT |  | Reverse | CGTGGCATCATAGAGAGCGT |
| *apoa2* | Forward | GCAGTTGAGCCAGCACTAGA | *lyz* | Forward | CAAGTGGTGTGATGACGGGA |
|  | Reverse | GCCAAACCACCAGCGATTTT |  | Reverse | CCTTTTACCCAGCGGGACAT |
| *ba1l* | Forward | TCCCGACAACTTCAGGCTTC | *zgc:56493* | Forward | TGTCAGAGCATCGCACCTTT |
|  | Reverse | ACCTGACGTTCATTGGCGAT |  | Reverse | CTACATCCTGTGCGTCGTCC |
| *actn3b* | Forward | TGCACTGACCTGAATGGCTT | *rplp2* | Forward | AAAAATGCGTTACGTGGCCG |
|  | Reverse | CTGCAGTGACATCCAGGGTT |  | Reverse | CATCATCAGCCTCGATCCCG |
| *hbaa1* | Forward | TGAGGTTCACGTGTCAGTCG | *rplp2l* | Forward | CACGCGAGACTCCTTCTCTC |
|  | Reverse | CCGCACAGTGTTGTTGTCAG |  | Reverse | CAGCCACGACCTTGCTCATA |
| *atp5fa1* | Forward | CAGAGACAACGGCAAACACG | *β-actin* | Forward | CGAGCAGGAGATGGGAACC |
|  | Reverse | GGATCCACCACCGAAGTTGT |  | Reverse | CAACGGAAACGCTCATTGC |

**Supplementary Table 3. Changes in transcriptional expression**

| Gene | 56μg poly(I:C) | Infected by *V. alginolyticus* | | Infected by *E. tarda* | |
| --- | --- | --- | --- | --- | --- |
|  |  | Live | Dying | Live | Dying |
| *apoa1* | ↑ | ↑ | - | ↑ | - |
| *tpma* | ↑ | ↑ | ↑ | ↑ | - |
| *mylipa* | - | - | - | - | ↓ |
| *calm2b* | ↑ | ↑ | - | ↑ | ↓ |
| *skp1* | - | - | ↓ | - | - |
| *crybb3* | ↑ | - | ↓ | ↑ | ↓ |
| *vat1* | - | - | - | - | - |
| *anp32e* | ↓ | ↑ | ↓ | ↑ | ↓ |
| *c1qbp* | ↓ | - | ↑ | - | ↓ |
| *apoa2* | ↑ | ↑ | - | ↑ | ↓ |
| *ba1* | - | - | ↓ | ↑ | ↓ |
| *actn3b* | - | ↑ | - | ↑ | - |
| *si:ch211-5k11.8* | ↑ | ↑ | - | ↑ | ↓ |
| *hbaa1* | ↑ | ↑ | ↓ | ↑ | ↓ |
| *atp5a1* | - | - | - | - | ↓ |
| *p4htm* | - | - | - | ↑ | ↓ |
| *gnb1a* | - | ↑ | - | ↑ | - |
| *actb2* | ↑ | - | - | - | - |
| *actb1* | - | - | - | - | - |
| *zgc:92533* | - | - | - | - | - |
| *fbxo2* | ↑ | ↑ | ↓ | ↑ | ↓ |
| *tnni2a.3* | ↑ | ↑ | ↓ | ↑ | ↓ |
| *cfl2l* | ↓ | - | - | - | ↓ |
| *lyz* | ↓ | ↑ | - | ↑ | ↓ |
| *zgc:56493* | - | - | - | - | - |
| *rplp2* | ↓ | - | - | - | - |
| *rplp2l* | ↓ | - | - | - | - |
